# Association of Subjective Sleep Pattern with Self-reported Diabetes in China

**DOI:** 10.21203/rs.3.rs-3196675/v1

**Published:** 2023-08-25

**Authors:** Lijing Yan, Huanhuan Sun, Yuling Chen, Xiaohui Yu, Jingru Zhang, Peijie Li

**Affiliations:** the Second Affiliated Hospital of Xi ‘an Jiaotong University; the First Affiliated Hospital of Xi’an Jiaotong University; the First Affiliated Hospital of Xi'an Jiaotong University; the First Affiliated Hospital of Xi ‘an Jiaotong University; the First Affiliated Hospital of Xi’an Jiaotong University; the First Affiliated Hospital of Xi’an Jiaotong University

**Keywords:** Subjective sleep duration, Self- reported diabetes, Lifestyle, Multilevel logistic regression, short-term

## Abstract

There is limited research investigating the relationship between self-reported diabetes mellitus and subjective sleep patterns. Our study aims to explore this association by analyzing trends in a cohort study conducted in China using data from the China Health and Nutrition Survey longitudinal research (CHNS). We used multilevel logistic regression models to analyze the relationship. Our findings indicate that the prevalence of self- reported diabetes in China increased from 1.10% in 2004 to 3.36% in 2015, with an increase in the prevalence of short-term sleep from 7.03–10.24%. The prevalence of self-reported diabetes increased with increasing BMI levels (Normal and below: 0.67–2.16%, Overweight: 1.58–4.35%, Obesity: 2.68–6.57%, p < 0.01). The short-term sleep subgroup had the highest prevalence (2.14–5.64%). Additionally, we found significant associations between age, education level, ethnicity, coffee, smoking, drinking and the self-reported diabetes. Interestingly, the risk ratios for self-reported diabetes differed between sleep durations. With 6–8hours as the reference group, the risk ratios for self-reported diabetes in the short-term, and long-term sleep subgroups were 1.80 (95% CI: 1.23–2.63), and 1.41 (95%CI: 1.01–1.96), respectively.

Raising awareness about the impact of irregular sleep duration on diabetes risk is essential, and these initiatives may serve as effective policies for diabetes control.

## 1 Introduction

Sleep is an essential behavior for maintaining biological rhythms and cellular repair [1]. According to Sheehan’s research, sleep duration might vary based on a person’s demand, which may be influenced by their ethnicity, region, cultural heritage, and lifestyle [2]. While sleep duration varies in different countries, it should be kept as adequate as possible to maintain individual physical and mental health. Recently, research on how sleep affects general health has expanded. Inappropriate sleep duration can pose a health risk to the body [3–6]. Although in some studies, sleep duration was recorded by some objective measures. Actigraphy has replaced Polysomnography (PSG) as the dominant tool in the study of sleep today. For studies with large populations, subjective sleep is still predominantly used due to the inaccessibility of equipment (Actigraphy or PSG). The existence of differences between subjective and objective sleep is then a matter of debate among scholars [7, 8]. Several researches have now concluded that the objective sleep duration results do not deviate from self-declared sleep duration by more than half an hour, and the correlation coefficient between the two is 0.70 [9–12]. It appears that the credibility of self-reported remains acceptable, especially for large cohort studies in medically deprived areas [13].

Globally, diabetes affects approximately 5 million [14]. However, China bears the greatest burden of this disease due to its large population size [15]. Given that early symptoms of diabetes are not readily apparent, blood testing is currently the only means available to identify and diagnose the condition. Consequently, early detection and screening for diabetes pose significant challenges. In epidemiological surveys, self-reported has emerged as a cost-effective approach with a high participation rate. Previous studies have confirmed the consistency between self-declared diagnoses, drug utilization patterns, and medical records [16]. Self-reported diabetes refers to patients being identified and informed of their condition by physicians using established diabetes diagnosis guidelines. While self-reported diabetes lacks objective measurement, it serves as a relatively reliable indicator of regional disparities in diabetes prevalence and undiagnosed cases, particularly in low- and middle-income areas where access to blood glucose testing and diagnosis is limited. It is worth noting that self-declared diabetes may underestimate the true prevalence due to the high occurrence of undiagnosed diabetes, but it remains a valid tool [17, 18]. Furthermore, compared to other chronic diseases, self-declared diabetes exhibits the highest sensitivity and specificity (kappa: 0.84 − 0.76) [19]. Its utilization in research is particularly advantageous in resource-constrained settings of low-income countries. Despite the limitations imposed by limited clinic access, self-reported diabetes generally aligns with the expected trends in the onset of diabetes. Moreover, when accurate data on the undiagnosed rate is unavailable, self-reported diabetes can serve as a corroborating indicator. Diabetes prevalence globally surpassed 9% in 2019, with a mere 49% of individuals diagnosed with the condition. Hence, self-reported diabetes accounts for approximately 4.5% of the average population. Due to medical constraints, this figure is even lower in middle- and low-income countries.

A considerable proportion of diabetic in China remain unaware of their condition, indicating the importance of diabetes awareness, particularly regarding symptoms and risk factors. Early detection through proactive medical counseling or seeking medical treatment has led to the identification of a significant percentage of newly diagnosed cases. Limited research has been conducted on the prevalence of self-reported diabetes and its influencing factors within diverse Chinese populations. Profiling the characteristics of self-reported diabetic patients can contribute to the promotion of diabetes health education. While some experimental studies have demonstrated that both excessively short and long sleep durations affect insulin and blood glucose levels, epidemiological studies have yielded conflicting conclusions regarding the eventual development of diabetes. [20] For instance, a study utilizing the NIH-AARP Diet and Health Cohort Study observed an increased diabetes risk in older adults with shorter sleep durations[21]. Conversely, another study conducted on elderly Chinese individuals reported contradictory findings. [22] Furthermore, the association between subjective sleep patterns and self-reported diabetes remains unknown, warranting further investigation. The objective of this investigation is to comprehensively examine the prevalence of self-reported diabetes mellitus in China, analyze its annual trend, and explore potential associations with subjective sleep patterns.

## 2 Materials and methods

### 2.1 Study Population

The CHNS is a stratified probability cohort study of the Chinese population that utilized a multistage, random cluster sampling strategy. It encompassed urban and rural areas in over 20 provinces and cities within China and included 15 ethnic groups, such as Man, Miao, Buyi, Tujia, and others. During the first stage of sampling, 2 cities and 4 counties were chosen in each province. In the second stage, a random selection of 2 urban and 2 suburban communities was conducted in each chosen city. In the third stage, a capital community and three rural villages were randomly selected in each chosen county, and 20 random families in each community were recruited. The CHNS commenced in 1989 and was tracked over nine waves in 1991, 1993, 1997, 2000, 2004, 2006, 2009, 2011, and 2015, with a survey population of almost 40,000 individuals overall. More information about the design, objectives, and survey methodology can be found on the CNHS homepage (https://www.cpc.unc.edu/projects/china). Since the earlier questionnaire did not have questions about sleep, our study was set to cover 2004 to 2015.

**Figure 1:**
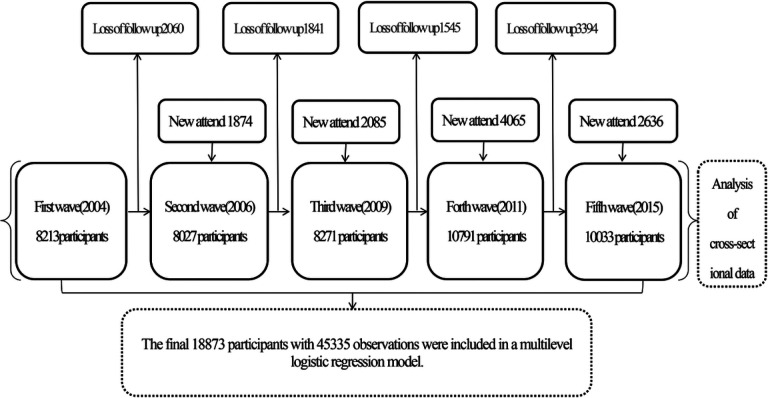
Flowchart for data collation and analysis

Our study enrolled participants who were between 18 and 65 years of age and completed questionnaires regarding their sleep duration and diabetes diagnosis. Since the CHNS was conducted at different times, there were constantly new enrolled attendees and participants who were lost of follow-up. We conducted a current prevalence analysis with different time perspectives as cross-sectional data and performed trend tests. Then, we performed multilevel logistic regression analysis from longitudinal data visualization (individuals as level 2 and each interview record as level 1). As shown in Fig. 1.

### 2.2 Outcomes and Covariates

In our study, we collected data on Total Sleep Time (TST) by asking participants, “How many hours each day do you usually sleep, including daytime and nighttime?” Participants recorded their answers in hours, which we divided into three sleep patterns: TST < 6 hours (short-term), 6–8 hours, and ≥ 9 hours (long-term) [5, 23, 24].

To measure the prevalence of self-reported diabetes, we asked participants, “Has a doctor ever told you that you suffer from diabetes?” Those who answered yes were classified as diabetic, while those who answered unknown or no were classified as non-diabetic.

We calculated Body Mass Index (BMI) by dividing the weight (kg) by the square of the height (m) based on physical measurements. We then graded BMI into three classifications according to the China obesity standard. BMI ≥ 28.0 was classified as obesity, BMI < 24.00 as normal or below, and overweight was situated between the two.

Covariates included in our study were age, sex (male, female), level of education (6–9 years for low, 10–12 for medium, and ≥ 13 for high, unknown), ethnicity (Han, other), residence area (urban, rural), tobacco use (yes or no), coffee and alcohol consumption (yes or no).

### 2.3 Statistical Analysis

First, we described the basic demographic characteristics, BMI classification, sleep patterns, and self-reported diabetes prevalence in the year of observation. We used means (standard deviations) for TST that conformed to a normal or approximately normal distribution, medians (quartile spacing) for age, weight, and height that were significantly skewed, and frequencies (percentages) for categorical variables.

Second, we analyzed the prevalence of self-reported diabetes among participants for gender, ethnicity, age group, residence, smoking, drinking, body mass index, and sleep pattern. We employed one-way ANOVA to analyze trends for TST across survey years and to compare TST between diabetics and non-diabetics. We used the Mann-Whitney Test to analyze trends for age, weight, and height across survey years. We also used the Cochran-Mantel-Haenszel Test to analyze trends for the different components of gender, residence areas, ethnicity, smoking, alcohol drinking, education, BMI, and sleep pattern across survey years. Additionally, the Cochran Mantel-Haenszel Test was used to analyze trends in the relationship between stratified variables and self-reported diabetes. And predict the incidence of self-reported diabetes using a linear model.

Third, we conducted a multilevel logistic regression model to assess the association of self-reported diabetes with sleep patterns, considering both within-individual and inter-individual sleep differences. We used self-reported diabetes as the response variable and different sleep patterns as the main independent variables, with adjustments made for age, gender, ethnicity, residence areas, BMI, education, smoking, coffee and alcohol in three models. We conducted statistical modeling and plotting using R4.2.1 with packages lme4 and ggplot2. We set p < 0.05 as the level of statistical significance in the study.

## 3 Results

In each of the five surveys, participants from rural areas (ranging from 59.25–66.98%) were overrepresented compared to those from urban areas. Moreover, the proportion of female participants (ranging from 51.50–53.11%) exceeded that of male participants in all surveys. The percentage of participants with a higher education level (≥ 13 years) increased from 4.19–15.36%. Coffee drinking also increased from 2.22 to 4.41%. In contrast, smoking and alcohol consumption rates decreased from 33.07–26.84% and 34.58–29.07%, respectively. The majority of participants slept for 6–8 hours at night (65.04–73.09%), while a minority of participants slept for a shorter duration (7.03–10.24%). Over the five waves of the survey, only the proportion of participants with a long-term sleep duration decreased year after year (from 27.93–15.97%). In contrast, the other sleep groups showed an upward trend (χ^2^ = 44.92, p < 0.01). The proportion of participants who were overweight or obese increased from 2004 to 2015 (χ^2^ = 341.37, p < 0.01).

**Figure 2:**
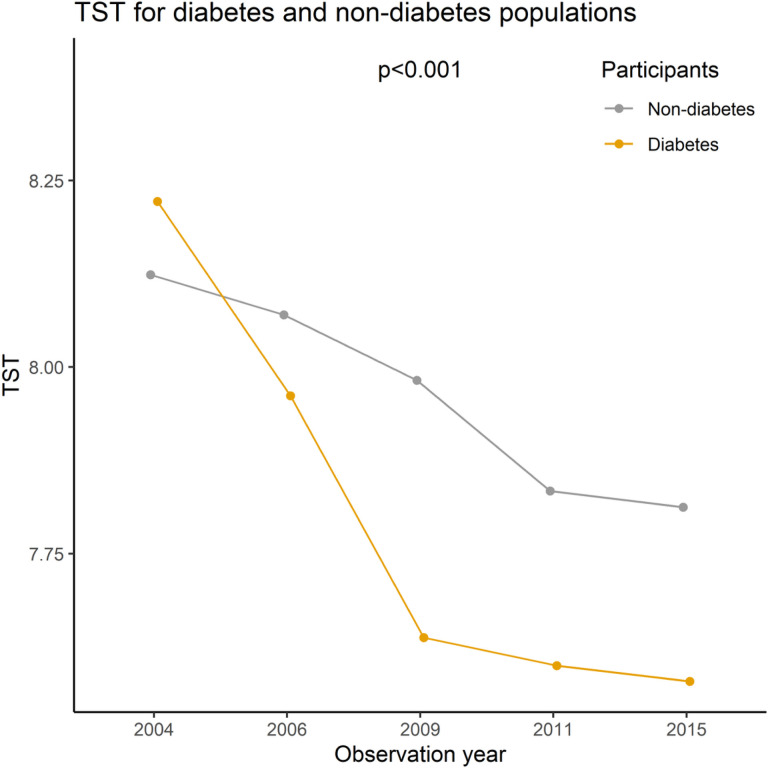
Mean TST for diabetes and non-diabetes in CHNS, 2004–2015

The prevalence of self-reported diabetes increased progressively from 1.11% in 2004 to 3.36% in 2015 (p < 0.01). The mean sleep duration also decreased gradually from 8.12 hours in 2004 to 7.8 hours in 2015 (F = 144.53, p < 0.01). This decrease was observed in both self-reported diabetes patients and self-reported non-diabetes participants, with sleep duration being lower in diabetes patients than in non-diabetes participants (p < 0.01) (see Fig. 2). The mean age of self-reported diabetes was 55.76. Overall, the prevalence of self-reported diabetes was slightly higher in males (1.10%–3.76%, p < 0.01), urban areas (1.77–4.48%, p < 0.01), Han ethnicity (1.16–3.60%, p < 0.01), no coffee (1.13–3.55%, p < 0.01), and non-drinking participants (1.30–3.33%, p = 0.01) than in their counterparts. The prevalence of self-reported diabetes also increased with increasing BMI levels (Normal and below: 0.67–2.16%, Overweight: 1.58–4.35%, Obesity: 2.68–6.57%, p < 0.01). The short-term sleep subgroup had the highest prevalence of self-reported diabetes among all waves (2.14–5.64%) and showed the maximum incremental increase (as shown in Table 2). After adjusting for the wave, different sleep subgroups were significantly correlated with self-reported diabetes rate (p < 0.01).

**Figure 3:**
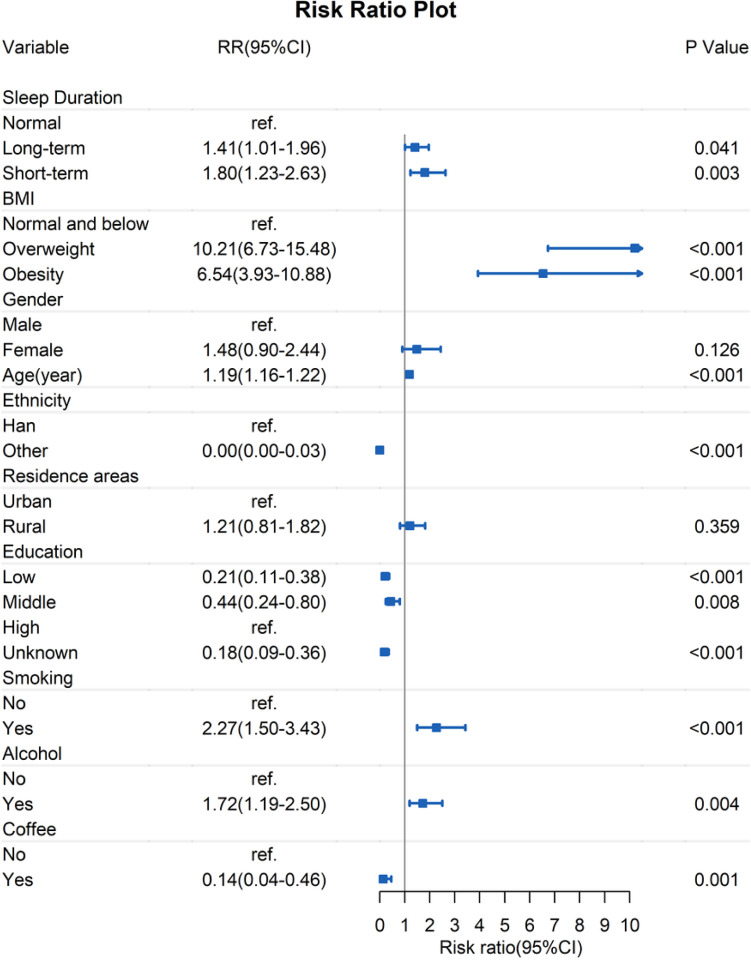
Multifactorial regression forest plot for self-reported diabetes

Taking sleep pattern as an independent risk factor for self-reported diabetes in Model 1, it was found the risk ratio for the short-term sleep subgroup was 2.22 (95%CI: 1.43–3.44, p < 0.001), and for the long-term sleep subgroup was 0.76 (95% CI: 0.52–1.11, p = 0.161) compared to the 6–8hours sleep subgroup. With the addition of BMI classification as a covariate in Model 2, a risk ratio for short sleep group was 2.02 (95% CI: 1.28–3.18, p = 0.002). In the final model, there were 18,873 participants with 45,335 observations, and we progressively added BMI classification, age, sex, ethnicity, education, coffee, smoking, drinking, and residence for model adjustment. After adjusting for all the covariates mentioned above, Model 3 showed that the risk ratio for the short-term sleep subgroup was 1.80 (95% CI: 1.23–2.63, p = 0.003) compared to the 6–8hours sleep subgroup. The risk ratios for the long-term sleep subgroup was 1.41 (95%CI: 1.01–1.96, p = 0.041). The results of the logistic regression models showed in Table 3. The final analysis showed short or long-term sleep, overweight or obese, age, smoking and drinking were positively associated with self-reported diabetes, ethnic minorities, coffee, and low education levels were negatively associated. (see Fig. 3).

## 4 Discussion

Our study presents compelling evidence that there was a consistent increase in the incidence of short-term sleep and self-reported diabetes in China between 2004 and 2015. Additionally, we have discovered a significant correlation between sleep patterns and self-reported diabetes. Specifically, participants in the short-term sleep subgroup were at a greater risk of self-reported diabetes compared to those in the 6–8hour sleep subgroup, while those in the long-term sleep groups was same.

The results of our survey indicate a declining trend in the average duration of sleep among Chinese residents over the years. However, debates exist regarding whether sleep duration for adults has decreased in the past decade [25]. Nevertheless, studies conducted in the Northeast of China suggest that visibly reduced sleep duration has become a pressing public health concern [26]. Furthermore, certain national disease control agencies have classified sleep deprivation as a public health epidemic [27]. In contrast to other studies [25, 28], our findings demonstrate a temporal shift in sleep duration from 2004 to 2015. The prevalence of long-term sleep duration decreased from 27.93% in 2004 to 15.97% in 2015, while the short-term sleep duration group increased from 7.03–10.24%.

Furthermore, we observed a tripling in the prevalence of self-reported diabetes between 2004 and 2015, rising from 1.10–3.37%. This trend aligns with other studies conducted in China, indicating a rapid increase in diabetes cases, leading to the nation having the highest number of patients globally [15, 29]. According to the results of some national-level surveys on diabetes prevalence and awareness, self-reported diabetes prevalence was estimated at 3.6% and 4.5% in 2013 and 2018, respectively [30] (Self-reported prevalence estimate = prevalence×awareness). This finding aligns with our results, indicating a steady rise in self-reported diabetes rates due to comprehensive primary healthcare efforts, approaching the global average(4.65%=9.3%×50%)[14]. Comparatively, the prevalence of self-reported diabetes among adults in Tehran was 4.98% in 2011, which exceeds our concurrent period [31]. A partial discrepancy with the findings of the 2020 Korean research may be due to the population base(8.97%=13.8%×65%)[32].Consequently, the burden imposed by diabetes presents a significant challenge for China in achieving global targets for non-communicable disease mortality and Sustainable Development Goals [33, 34]. Therefore, further implementation of public policies and preventive measures for diabetes is recommended.

Although there may be slight variations in the definitions of diabetes and sleep duration across different studies, our research demonstrates a clear association between abnormal sleep patterns and an increased risk of self-reported diabetes. Specifically, we have found that both short and long sleep durations contribute to this risk, with long sleep duration being particularly significant when considering all relevant factors in our model. This finding is consistent with a comprehensive 2-year follow-up study involving a cohort of 100,000 individuals, led by Dr. Qiaofeng Song. Within this study, participants who slept more than 8.5 hours per night were found to have a higher risk of developing diabetes, even after controlling for other variables (HR, 1.37; 95% CI, 1.03–1.81)[35].

Our findings align with some authors’ analysis, which also indicates that individuals with abnormal sleep durations are at a greater risk of type 2 diabetes compared to those with normal sleep durations[36, 37]. Significant, the risk is twice as high for individuals with short sleep durations and 1.6 times as high for those with long sleep durations. Similarly, our study identified a self-reported diabetes risk of 1.4 among individuals with long-term sleep durations (≥ 9 hours). Moreover, the analysis revealed that individuals with short-term sleep durations had an even higher predicted risk of self-reported diabetes, regardless of the inclusion of covariates (OR: 1.8–2.22).

While some controversy exists regarding the causal relationship between prolonged sleep and diabetes, it is worth noting that this association appears to be more significant within certain populations. For example, a Korean study following Asians over a period of 16 years found a significant association between long sleep duration and diabetes, but only among obese [38]. Similarly, a Chinese cohort study indicated that this association was particularly pronounced among middle-aged and elderly individuals.

Furthermore, our study identified lower rates of self-reported diabetes among ethnic minorities and individuals with lower levels of education. This finding may be attributed to healthcare inequities, as Sarah’s study on the prevalence of self-reported diabetes in the United States suggests that ethnic minorities and individuals with lower education levels are generally less aware of diabetes and have limited access to healthcare[39]. Additionally, the prevalence of self-reported diabetes was found to be high among overweight and obese individuals, highlighting the role of diabetes awareness as a significant influencing factor[40].

In conclusion, our findings indicate a significant increase in the prevalence of self-reported diabetes and short-term sleep duration among Chinese adults over the past decade. Furthermore, our analysis revealed that short-term or long-term sleep was associated with an increased risk of self-reported diabetes. In light of these findings, it is important to raise awareness about the impact of abnormal sleep duration on the risk of developing diabetes. Such initiatives could potentially serve as effective policy measures for diabetes control.

There are several limitations in our study that should be acknowledged. Firstly, the self-reported nature of either sleep duration or diabetes in the survey introduces the possibility of recall bias during the interview. However, previous studies have reported the reliability and accuracy of self-reported measures when compared to objective measurements. In addition, self-reported remains a cost-effective approach with a higher participation rate. Secondly, the year of observation was not a fixed time interval, which may have resulted in an inadequate assessment of time effects. Thirdly, to better understand the relationship between sleep duration and diabetes, a dose-effect analysis should be conducted in future studies.

## Figures and Tables

**Table 1:** Characteristics of Participant in CHNS, 2004–2015

	Observation year					Test statistic
All participants	2004 (n = 8213)	2006 (n = 8027)	2009 (n = 8271)	2011 (n = 10791)	2015 (n = 10033)	
Sex(%)						7.58 (0.11)¢
Male	3983 (48.50)	3864 (48.14)	3980 (48.12)	5081 (47.09)	4704 (46.89)	
Female	4230 (51.50)	4163 (51.86)	4291 (51.88)	5710 (52.91)	5329 (53.11)	
Age, Mean(SD)	43.19 (12.06)	44.22 (11.92)	44.98 (12.16)	45.94 (12.14)	47.07 (12.02)	579.23 (<0.01)
Weight, Median (IQR)	59.50 (14.40)	60.00 (14.20)	60.40 (14.60)	62.00 (15.00)	62.80 (15.70)	558.47 (<0.01)
Height, Median (IQR)	161.00 (12.10)	161.20 (12.00)	161.50 (12.00)	162.00 (12.10)	161.80 (12.00)	53.90 (< 0.01)
TST, Mean(SD)	8.12 (1.25)	8.08 (1.23)	7.97 (1.16)	7.82 (1.18)	7.80 (1.13)	144.53 (<0.01)
Residence areas (%)						193.43 (< 0.01) ¢
Urban	2773 (33.76)	2651 (33.02)	2786 (33.68)	4397 (40.75)	3838 (38.25)	
Rural	5440 (66.24)	5376 (66.98)	5485 (66.32)	6394 (59.25)	6195 (61.75)	
Education(%)						389.44 (<0.01) ¢
Low	4837 (59.89)	4277 (53.28)	4658 (56.32)	5390 (49.95)	4921 (49.05)	
Middle	1830(22.28)	1860(23.17)	1785(21.58)	2456(22.76)	2469(24.61)	
High	344(4.19)	480(5.98)	493(5.96)	1452(13.46)	1541(15.36)	
Unknown	1202(14.64)	1410(17.57)	1335(16.14)	1493(13.84)	1102(10.98)	
Smoking(%)	2716(33.07)	2547(31.73)	2615(31.62)	3289(30.48)	2693(26.84)	99.29(< 0.01) ¢
Alcohol drinking (%)	2840(34.58)	2716(33.84)	2938(35.52)	3902(36.16)	2917(29.07)	140.53(<0.01) ¢
Coffee(%)	182(2.22)	193 (2.40)	272 (3.29)	763(7.07)	442 (4.41)	392.60(<0.01)
Ethnicity												
Han	7168(87.28)	7023(87.49)	7225(87.35)	9773(90.57)	8966(89.37)	83.65(< 0.01) ¢
Other	1045(12.72)	1004(12.51)	1046(12.65)	1018(9.43)	1067(10.63)	
BMI (%)						341.37(< 0.01) ¢
Normal and below	4959(60.38)	4732(58.95)	4686(56.66)	5706 (52.88)	4359 (43.45)	
Overweight	2150(26.18)	2194(27.33)	2400(29.02)	3443 (31.91)	3123 (31.13)	
Obesity	598(7.28)	601 (7.49)	753 (9.10)	1283 (11.89)	1309 (13.05)	
Missing	506(6.16)	500(6.23)	432(5.22)	359(3.33)	1242(12.38)	
Sleep Duration (%)						44.92 (< 0.01) ¢
Short-term	577(7.03)	584(7.28)	684(8.27)	1192(11.05)	1028(10.24)	
6–8hours	5342(65.04)	5325(66.34)	5766(69.71)	7564 (70.10)	7403(73.79)	
Long-term	2294(27.93)	2118(26.39)	1821(22.02)	2035(18.85)	1602(15.97)	

Mann-Whitney Test

¢ Cochran-Mantel-Haenszel Test

One-way ANOVA

**Table 2:** Prevalence of self-reported diabetes in CHNS, 2004–2015

		Cross-sectional data analysis for five waves					Cochran-Mantel-Haenszel Test (CMHx^2^) (*p*)
		2004	2006	2009	2011	2015	
Self-repoted diabetes (%)		91(1.11)	105(1.31)	172(2.08)	332(3.08)	337(3.36)	168.76 (< 0.01)
Age	55.76(7.25)	55.49(7.03)	53.78(7.41)	55.10(7.25)	55.72(7.56)	56.81(6.80)	
Ethnicity	CMH**x**^2^ (*p*)	1.28(0.26)	3.32(0.07)	0.76(0.38)	6.45(0.01)	15.41 (< 0.01)	24.03(<0.01)
	Han (%)	83(1.16)	98(1.40)	154(2.13)	314(3.21)	323(3.60)	
	Other (%)	8(0.77)	7(0.70)	18(1.72)	18(1.77)	14(1.31)	
Gender	CMH**x**^2^ (*p*)	0.001(0.98)	1.15(0.28)	4.82(0.03)	0.73(0.39)	4.45(0.03)	8.49 (< 0.01)
	Male(%)	44(1.10)	56(1.45)	97(2.44)	164(3.23)	177(3.76)	
	Female(%)	47(1.11)	49(1.18)	75(1.75)	168(2.94)	160(3.00)	
Residence areas	CMH**x**^2^ (*p*)	16.59 (< 0.01)	37.51 (< 0.01)	38.51 (< 0.01)	25.74(<0.01)	24.13(<0.01)	127.88(<0.01)
	Urban(%)	49(1.77)	64(2.41)	96(3.45)	180(4.09)	172(4.48)	
	Rural(%)	42(0.77)	41 (0.76)	76(1.39)	152(2.38)	165(2.66)	
Education	CMH**x**^2^ (*p*)	6.25(0.10)	7.79(0.05)	0.92 (0.82)	1.15(0.76)	13.90(<0.01)	6.06 (0.11)^§^
	Low(%)	44(0.91)	46(1.08)	95(2.04)	166(3.08)	168(3.41)	
	Middle(%)	22(1.20)	25(1.34)	38(2.13)	82(3.34)	96(3.89)	
	High(%)	7(2.03)	12(2.50)	13(2.64)	40(2.75)	29(1.88)	
	Unknown(%)	18(1.50)	22(1.56)	26(1.95)	44(2.95)	44(3.99)	
Smoking	CMH**x**^2^ (*p*)	0.06(0.81)	0.02(0.89)	0.07(0.79)	0.001(0.98)	1.42(0.23)	0.53(0.47)
	Yes(%)	29(1.07)	34(1.33)	56(2.14)	101(3.07)	100(3.71)	
	No(%)	62(1.13)	71(1.30)	116(2.05)	231(3.08)	237(3.23)	
Alcohol drinking	CMH**x**^2^ (*p*)	5.43(0.02)	3.91(0.05)	2.15(0.14)	1.95(0.16)	0.06(0.81)	6.86(0.01)
	Yes(%)	21 (0.74)	26(0.96)	52(1.77)	108(2.77)	100(3.43)	
	No(%)	70(1.30)	79(1.49)	120(2.25)	224(3.25)	237(3.33)	
BMI	CMH**x**^2^ (*p*)	27.36(<0.01)	35.19(<0.01)	54.59(<0.01)	98.84(<0.01)	64.66(<0.01)	274.89(< 0.01)^Ψ^
	Normal and below(%)	33(0.67)	37(0.78)	57(1.22)	100(1.75)	94(2.16)	
	Overweight(%)	34(1.58)	42(1.91)	74(3.08)	143(4.15)	136(4.35)	
	Obesity(%)	16(2.68)	20(3.33)	36(4.78)	85(6.63)	86(6.57)	
Coffee	CMH**x**^2^ (*p*)	2.09(0.15)	2.62(0.11)	2.09(0.15)	12.84(<0.01)	0.002(0.97)	6.88(<0.01)
	Yes(%)	0(0.00)	0(0.00)	9(3.31)	7 (0.92)	15(3.39)	
	No(%)	91 (1.13)	105(1.34)	163(2.04)	325(3.24)	247(3.55)	
Sleep Duration	CMH**x**^2^ (*p*)	10.23(<0.01)	2.73(0.26)	15.52(<0.01)	8.79(0.01)	16.87(<0.01)	43.59(<0.01)
	Short-term(%)	12(2.08)	12(2.05)	27(3.95)	53(4.45)	57(5.54)	
	6–8hours(%)	46(0.86)	67(1.26)	119(2.06)	224(2.96)	231(3.12)	
	Long-term(%)	33(1.44)	26(1.23)	26(1.43)	55(2.71)	49(3.06)	

§ The unknown group was excluded

Ψ The missing group was excluded

**Table 3:** Association between self-reported diabetes and sleep duration in CHNS

	Model1		Model2*		Model3^#^	
Sleep Duration	RR(95%CI)	*p*	RR(95%CI)	*p*	RR(95%CI)	*p*
Short-term	**2.22(1.43–3.44)**	**<0.001**	**2.02 (1.28–3.18)**	**0.002**	**1.80(1.23–2.63)**	**0.003**
6–8hours	ref.		ref.		ref.	
Long-term	0.76(0.52–1.11)	0.161	0.73(0.49–1.09)	0.121	**1.41(1.01–1.96)**	**0.041**

* adjusting for the BMI.

# adjusting for the age(continuous), gender, ethnicity, residence areas, education, smoking, alcohol, coffee and BMI.

## Data Availability

The datasets analyzed during the current study are available in the China Health and Nutrition Survey repository (http://www.cpc.unc.edu/projects/china).
